# Supplementary material for: Reconciling Mining with the Conservation of Cave Biodiversity: A Quantitative Baseline to Help Establish Conservation Priorities
Source: PLoS One. 2016 Dec 20;11(12):e0168348. doi: 10.1371/journal.pone.0168348 (PMC5173368; doi:10.1371/journal.pone.0168348)
Supplement: S1 Dataset — (ZIP) [file pone.0168348.s002.zip › Taxa/Serra Norte/SN_2013/N4E/Lista N4E-44.pdf]

CAVIDADE N4E-0044							
**Classe**	**Ordem**	**Fam/Outros**	**Gên/Outros**	**Espécie**	**S-U**	**U-S**	**Zon.**
Arachnida	Acari	Ixodidae	*Amblyomma*	sp.1		X	P
Arachnida	Acari	Neothyridae	*Diplothyrus*	jovem		X	P
Arachnida	Acari	Trombiculidae		sp.39		X	P
Arachnida	Amblypygi	Charinidae	*Charinus*	sp.1	1	2	E/P
Arachnida	Amblypygi	Phrynidae	*Heterophrynus*	*longicornis*	1	2	E/P
Arachnida	Araneae	Corinnidae		jovem		1	P
Arachnida	Araneae	Ctenidae	*Ctenus*	sp.1	2		
Arachnida	Araneae	Mimetidae	*Ero*	sp.1	X		E
Arachnida	Araneae	Mysmenidae	*Microdipoena*	sp.1	X		P
Arachnida	Araneae	Ochyroceratidae	*Ochyrocera*	sp.1	X	X	E/P
Arachnida	Araneae	Ochyroceratidae	*Speocera*	sp.1	X	X	P
Arachnida	Araneae	Oonopidae		jovem	X		P
Arachnida	Araneae	Pholcidae	*Leptopholcus*	sp.1	X		E/P
Arachnida	Araneae	Pholcidae	*Mesabolivar*	*aurantiacus*	X		
Arachnida	Araneae	Pholcidae	*Mesabolivar*	sp.1		X	P
Arachnida	Araneae	Prodidomidae		jovem		X	P
Arachnida	Araneae	Salticidae		jovem		X	P
Arachnida	Araneae	Theridiidae	*Theridion*	sp.1	X		E
Arachnida	Araneae	Theridiidae	*Theridion*	sp.2		X	P
Arachnida	Araneae	Theridiosomatidae	*Plato*	sp.1	X	X	E/P
Arachnida	Opiliones	Escadabiidae		sp.2	X		P
Arachnida	Pseudoscorpiones	Chernetidae	*Spelaeochernes*	sp.1	X	X	P
Arachnida	Schizomida	Hubbardiidae	aff. *Rowlandius*	sp.1		X	P
Chilopoda	Scolopendromorpha	Scolopocryptopidae		jovem		1	P
Chilopoda	Scutigeromorpha	Pselliodidae		jovem	1		E
Diplopoda	Polydesmida	Pyrgodesmidae		sp.1		X	P
Entognatha	Collembola	Entomobryidae		sp.1	X	X	P
Entognatha	Collembola	Isotomidae		sp.1		X	P
Entognatha	Collembola	Paronellidae		sp.1		X	P
Entognatha	Collembola	Paronellidae		sp.4		X	P
Entognatha	Diplura	Campodeidae		sp.1	X	X	P
Entognatha	Diplura	Projapygidae		sp.1	X	X	P
Insecta	Blattodea			jovem		1	P
Insecta	Coleoptera			jovem	X	X	E/P
Insecta	Diptera	Limoniidae		sp.		X	P
Insecta	Diptera	Psychodidae	*Breviscapus*	sp.		X	P
Insecta	Diptera	Psychodidae	Phlebotominae	sp.		X	P
Insecta	Diptera	Tipulidae	Tipulinae	sp.	X		E
Insecta	Homoptera	Cixiidae		sp.1		X	P
Insecta	Homoptera	Cixiidae		sp.5	5		P
Insecta	Hymenoptera	Formicidae	*Brachymyrmex*	sp.1	X		P
Insecta	Hymenoptera	Formicidae	*Camponotus*	sp.1	X	X	P
Insecta	Hymenoptera	Formicidae	*Camponotus*	sp.2	X	X	E/P
Insecta	Hymenoptera	Formicidae	*Eurhopalothrix*	sp.1	X	X	P
Insecta	Hymenoptera	Formicidae	*Gnamptogenys*	sp.1		X	P
Insecta	Hymenoptera	Formicidae	*Pachycondyla*	sp.1		X	P
Insecta	Hymenoptera	Formicidae	*Pachycondyla*	sp.2		X	P
Insecta	Hymenoptera	Formicidae	*Paratrechina*	sp.1		X	P
Insecta	Hymenoptera	Formicidae	*Pheidole*	sp.1	X		P

Insecta	Hymenoptera	Formicidae	*Pheidole*	sp.3		X	P
Insecta	Hymenoptera	Formicidae	*Solenopsis*	sp.1		X	P
Insecta	Hymenoptera	Formicidae	*Trachymyrmex*	sp.1		X	P
Insecta	Isoptera			sp.	X	X	E/P
Insecta	Lepidoptera	Noctuoidea		sp.2		9	
Insecta	Orthoptera	Gryllidae	Nemobiinae	sp.		2	
Insecta	Orthoptera	Phalangopsidae	*Paraclodes*	sp.	1		
Insecta	Orthoptera	Phalangopsidae	*Phalangopsis*	sp.	1	55	P
Insecta	Psocoptera	Epipsocidae	*Epipsocus*	sp.2	X	X	P
Gastropoda	Pulmonata	Subulinidae		sp.2		X	P
Gastropoda	Pulmonata	Systrophiidae	*Happia*	sp.		X	P
Malacostraca	Isopoda	Dubioniscidae		sp.1		X	P
Symphyla		Scutigerellidae		sp.		X	P
Turbellaria	Tricladida			sp.2		1	P
Amphibia	Anura	Strabomantidae	*Pristimantis*	cf. *fenestratus*	7	2	E/P
Mammalia	Chiroptera	Emballonuridae	*Peropteryx*	*kappleri*	1		E
Reptilia	Squamata	Gekkonidae	*Thecadactylus*	sp.	1		P
